# *CYP24A1* and *TRPC3* Gene Expression in Kidneys and Their Involvement in Calcium and Phosphate Metabolism in Laying Hens

**DOI:** 10.3390/ani14101407

**Published:** 2024-05-08

**Authors:** Letícia Alves Salmória, Adriana Mércia Guaratini Ibelli, Fernando Castro Tavernari, Jane Oliveira Peixoto, Marcos Antônio Zanella Morés, Débora Ester Petry Marcelino, Karine Daenquele Silva Pinto, Arlei Coldebella, Diego Surek, Vicky Lilge Kawski, Mônica Corrêa Ledur

**Affiliations:** 1 Programa de Pós-Graduação em Ciências Veterinárias, Universidade Estadual do Centro-Oeste, Guarapuava 85015-430, PR, Brazil; letiasalmoria3@gmail.com (L.A.S.); jane.peixoto@embrapa.br (J.O.P.); 2 Embrapa Suínos e Aves, Concórdia 89715-899, SC, Brazil; fernando.tavernari@embrapa.br (F.C.T.); marcos.mores@embrapa.br (M.A.Z.M.); arlei.coldebella@embrapa.br (A.C.); diego.surek@embrapa.br (D.S.); vicky.kawski@embrapa.br (V.L.K.); 3 Programa de Pós-graduação em Zootecnia, Centro de Educação Superior do Oeste (CEO), Universidade do Estado de Santa Catarina, UDESC, Chapecó 89815-630, SC, Brazil; 4 Faculdade de Concórdia, Concórdia 89700-00, SC, Brazil; deboraester.agro@gmail.com; 5 Departamento de Zootecnia, Universidade Federal de São João del Rei, São João del Rei 36301-360, MG, Brazil; karine.daenquele@yahoo.com

**Keywords:** mineral, egg quality, qPCR, chickens

## Abstract

**Simple Summary:**

Calcium and phosphorus are essential minerals required for animal health, especially in laying hens since they are important components in bone and eggshell formation. Diets with inadequate calcium and phosphorus can interfere with their absorption and digestibility, resulting in eggshell quality losses and reduced productive life, affecting egg production and welfare. Although mineral metabolism has been widely studied in young laying hens, the mechanisms of absorption, reabsorption, and mineral homeostasis of laying hens with advancing age are not well known. Therefore, we studied eight genes involved in Ca and P metabolism and observed that two of them (*CYP24A1* and *TRPC3*) showed differential expression according to the Ca/P ratios provided in the diet. These results contribute to understanding how Ca and P metabolism are regulated in hens during their late laying cycle, allowing the development of new nutritional strategies to improve hens’ productive life as well as their egg quality.

**Abstract:**

Ca and P homeostasis across the egg-laying cycle is a complex process involving absorption in the small intestine, reabsorption/excretion in the kidneys, and eggshell gland secretion. Diets with inadequate calcium and phosphorus can interfere with their absorption and digestibility, resulting in eggshell quality losses and reduced productive life, affecting egg production and welfare. A better understanding of gene expression profiles in the kidneys of laying hens during the late egg-laying period could clarify the renal role in mineral metabolism at this late stage. Therefore, the performance, egg quality and bone integrity-related traits, and expression profiles of kidney candidate genes were evaluated in 73-week-old laying hens receiving different Ca and P ratios in their diet: a high Ca/P ratio (HR, 22.43), a low ratio (LR, 6.71), and a medium ratio (MR, 11.43). The laying hens receiving the HR diet had improved egg production and eggshell quality traits compared to the other two groups. Humerus length was shorter in the HR than in the other groups. The *CYP24A1* and *TRPC3* genes were differentially expressed (*p.adj* ≤ 0.05) among the groups. Therefore, their expression profiles could be involved in calcium and phosphate transcellular transport in 73-week-old laying hens as a way to keep mineral absorption at adequate levels.

## 1. Introduction

In laying hens, after the peak of production (from the 23rd to 32nd week), the egg production and shell quality tend to decrease with age [1,2]. Different factors are associated with this process, and the reduction in body mineral absorption is considered one of the most important, especially when inadequate levels of minerals are provided in the diet [3,4]. In the young phase, the mineral retention rate is about 60%, and with aging the absorption can be reduced by up to 40% [5]. Calcium (Ca) and phosphorus (P) are considered essential minerals for bone structure and eggshell formation, and the reduced absorption of Ca and P with aging directly affects these traits [6]. In addition, the maintenance of Ca and P homoeostasis across the egg-laying cycle is a very complex process involving absorption in the small intestine, reabsorption/excretion in the kidneys, medullary bone dissolution, and eggshell gland secretion [7]. Furthermore, several factors can influence the mineral metabolism related to the phenotypic traits of laying hens, such as mineral granulometry, time of mineral absorption by the intestine, mineral source (organic or inorganic), and intestinal pH, among others [8,9,10,11]. In the last years, it has been demonstrated that an increase in calcium granulometry leads to better mineral use by hens, which has become a favorable alternative in poultry production [12], since an improvement in eggshell quality traits has been shown [13,14].

Among the tissues involved in mineral metabolism, the intestine and bones simultaneously participate in the mineral regulatory process through hormonal regulators, such as parathyroid hormone (PTH), estrogen, calcitonin, vitamin D, FGF23 (Fibroblast growth factor 23), and membrane receptors [15,16]. Moreover, the involvement of cytokines and inflammatory mediators in mineral homeostasis has also been described [16]. Glomerular filtration, tubular reabsorption, and tubular secretion, which occur in the kidneys, have been considered the main mechanisms of mineral regulation in laying hens for decades [17]. These organs are fundamental for homeostasis, adjusting the Ca and P amounts retained, and modulating their absorption and reabsorption in the nephrons [18,19]. Furthermore, the kidneys have a key role in the production of the bioactive form of vitamin D [20], which is required for mineral homeostasis and improved egg quality [21,22].

In the last years, there has been an increase in studies trying to understand the impact of different diets on gene expression profiles and their impact on animal metabolism [23,24,25]. High expression levels of the vitamin D receptor (*VDR)* and calbindin-D28k (*CaBP-D28k*) genes in the duodenum, and *CaBP*-*D28k,* ATPase Plasma Membrane Ca2+ Transporting 1 (*PMCA1b*), and fibroblast growth factor receptor 1 (*FGFR1*) in the kidneys, were associated with Ca and P absorption when laying hens at the peak of production were compared to hens in their late egg-laying period [7]. Other studies have been conducted evaluating different mineral levels and their influence on bone and production traits in young laying hens and the impact of candidate genes in mineral absorption and homeostasis [26,27]. However, there is a lack of information on the response of hens in their late laying cycle to different levels of Ca and P in the diet, as well as how gene expression is regulated to improve the use of nutrients. Moreover, the optimization of the Ca/P ratio in the diet across the whole production cycle would guarantee high performance levels, eggshell quality, and bone integrity, preventing osteoporosis. Therefore, in the current study, egg production, egg quality and bone-related traits, and the expression profile of candidate genes in the kidney were evaluated in 73-week-old laying hens fed with three different Ca/P ratios in the diet. Eight candidate genes were chosen according to previous studies reporting their functions associated with calcium and phosphorus homeostasis to be evaluated in the kidneys: *CYP24A1* (cytochrome P450 family 24 subfamily A member 1), *FGF3R* (member of the basic fibroblast growth factor), *PTH* (parathyroid hormone), *TRPV6* (transient receptor potential cation channel subfamily V member 6), *CALB1* (calbindin 1), *CALM2* (calmodulin 2), *FGF1* (fibroblast growth factor 1), and *TRPC3* (transient receptor potential cation channel subfamily C member 3) [28,29,30,31]. A better understanding of gene expression profiles in the kidneys of laying hens during the late egg-laying period could clarify the renal role in the mineral metabolism at this late stage and point to nutritional strategies for improving the persistence of egg production, farm economy, and laying hens’ welfare.

## 2. Materials and Methods

### 2.1. Animals, Phenotypic Traits, and Sample Collection

This study followed the guidelines of the National Council for the Control of Animal Experiments (CONCEA) and Ethics Committee for Animal Use (CEUA) of the Embrapa Swine and Poultry National Research Center, with approval protocol #017/2016. This study was carried out with commercial Bovans White laying hens of up to 72 weeks of age in a randomized block design with a total of three treatments with 10 replicates of 7 birds per cage, totaling 70 hens. A standard diet [32] was given to the hens during the first 23 weeks of age, and from 24 to 72 weeks of age they received the experimental diet, totaling 48 weeks of experiment. The experimental diets were high Ca/P ratio (HR): 22.43 Ca/P ratio, with 4.71% Ca and 0.21% P; low Ca/P ratio (LR): 6.71 Ca/P ratio, with 3.29% Ca and 0.49% P; and medium Ca/P ratio (MR): 11.43 Ca/P ratio, containing 4% Ca and 0.35% P (Supplementary Table S1). Except for Ca and P, all diets were formulated according to Rostagno et al. [32], and the calcium granulometry used for feed composition was 50% fine and 50% coarse.

Performance and egg quality evaluation was carried out every 28 days for the following traits:

- Feed intake was determined at the end of each 28-day period by dividing the amount of feed consumed in each experimental unit by the number of birds in the experimental units per day (corrected for mortality).
- Egg production was collected daily and measured as percentage and per hen housed. The average egg weight was obtained by dividing the total weight of eggs collected in the last 5 days of each of the 28-day periods by the number of eggs collected, per experimental unit.
- Egg mass was expressed in g per bird per day (g/bird/day) and obtained by multiplying the average egg weight in the period by the total number of eggs produced in the respective period, divided by the total number of birds on the days relating to that period.
- Feed conversion was obtained by dividing feed intake by the production in dozen of eggs (kg/dz), or the production in kg of eggs (kg/kg), in each period.
- To measure the egg components, the yolk, albumen, and shell were evaluated using six eggs from each replicate, collected randomly and daily from the total number of eggs collected in the last three days of each period. The eggs from each replicate and each day were weighed individually, broken, and their yolk and shell weighed. To separate the egg components (yolk, shell, and albumen), a conventional yolk separator was used.
- The shells were washed, left to dry, and weighed.
- The albumen weight was obtained as the difference between the total egg weight and the shell and yolk weights. The yolk, albumen, and shell weights were determined as percentages. The egg tester (DET6500; Nabel, Kyoto, Japan) was used to evaluate albumen height, yolk color, Haugh unit [33], shell strength, and shell thickness.

At 73 weeks of age, 30 chickens (10/group) were sampled and transferred to the necropsy facility at the Embrapa Swine and Poultry National Research Center where they were weighed and euthanized by cervical dislocation for kidney, humerus, and tibia sample collection. We collected samples from the caudal lobe of the left kidney in all hens, which were immediately stored in liquid nitrogen and subsequently kept at −70 °C for gene expression analysis and stored in 4% paraformaldehyde for histological analysis. Bones were cleaned and stored at −20 °C for morphological and physical analysis.

### 2.2. Kidney Histological Analysis

Samples were dehydrated in crescent ethanol concentration, diaphanized with xylol, and then embedded in paraffin. Sections of the tissues were cut in an automatic microtome with 2 to 5 μm thickness and stained with hematoxylin and eosin protocol. Slides were visualized by light microscopy with an Olympus BX53 (Olympus, Tokyo, Japan) microscope with a 10× eyepiece and 5× to 50× objectives for routine histopathological analysis. The percentage of samples presenting lesions was calculated in relation to the total of samples analyzed in each group. Images corresponding to samples from the three studied groups were captured by microscope camera (AXIO Scope AI; Carl Zeiss, Gottingen, Germany) in the 20× and 50× objectives.

### 2.3. Tibia and Humerus Morphological and Physical Analysis

The tibia and humerus were weighed on a precision scale, their length was measured with a paquimeter, and they were stored at −20 °C for further analyses. Tibias were defrosted and kept at 4 °C for 24 h, when they were dissected for the evaluation of the following integrity-related traits: breaking strength (BS, kgf), flexibility (FLEX, kgf/mm), and Seedor Index (SI). The bone resistance analyses were performed in the texturometer TA–XTPlus Texture Analyzer (Texture Technologies Corporation, Hamilton, MA, USA), using the 3-Point Bending Rig (HDP/M3PB) with 5 kg load cell Heavy-Duty Platform (HDP/90), according to Cruz et al. [34]. Bones were positioned with their epiphyses resting on two supports spaced at 40 mm. The BS is the maximum stress that the bone can withstand while being stretched before breaking, and the FLEX is the ratio between the BS and the bone elastic linear displacement. The Seedor Index (SI), a measure of bone density, was calculated by the ratio between tibia weight and length [35].

### 2.4. RNA Extraction, Quantification, and Complementary DNA (cDNA) Synthesis

A total of 100 mg of kidney samples was macerated in liquid nitrogen, and the total RNA extraction was performed using Trizol reagent (Life Technologies, Carlsbad, CA, USA), according to the manufacturer’s instructions, followed by purification on a silica column with Qiagen RNEasy kit (Qiagen, Hilden, Germany). RNA concentration was measured with the Biodrop device (Biochrom, Holliston, MA, USA) using 1 µL of each sample. The A260/A280 ratio was used to assess RNA purity, which was considered adequate in the range of 1.9 to 2.1.

The cDNA synthesis was performed with the GoScript Reverse Transcriptase kit (Promega, Madison, WI, USA) using 3 μg of total RNA, following the manufacturer’s recommendations for poly-A selection. The cDNA was diluted 10 times in ultrapure nuclease-free water (~15 ng/uL) and stored at −20 °C for subsequent qPCR analyses.

### 2.5. Reference Gene Selection

Ten candidate genes widely used as references were chosen to be evaluated according to their stability to be used as normalizers in the gene expression analysis from the kidney tissue: *GAPDH* (Glyceraldehyde 3 Phosphate Dehydrogenase), *HMBS* (Hydroxymethylbilane Synthase), *HPRT1* (Hypoxanthine Phosphoribosyltransferase 1), *MRPS27* (Mitochondrial Ribosomal Protein S27), *MRPS30* (Mitochondrial Ribosomal Protein S30), *RPL30* (Ribosomal Protein L30), *RPL4* (Ribosomal Protein L4), *RPL5* (Ribosomal Protein L5), *RPLP1* (Ribosomal Protein Lateral Stalk Subunit P1), and *TOP2B* (DNA Topoisomerase II) (Table 1). The qPCR reactions were performed with QuantStudio 6 Real-Time PCR equipment (Life Technologies, Carlsbad, CA, USA) using the following protocol: 1X GoTaq qPCR Master Mix (Promega, Madison, WI, USA), 0.133 uM forward primer, 0.133 uM reverse primer, cDNA in 1:10 dilution, and ultrapure water (Promega, Madison, WI, USA) up to 15 μL of the final volume. All reactions were performed in duplicate, using negative controls to detect possible contamination. Cycling conditions were 95° for 2 min and 40 cycles of 15 s at 95 °C and 30 s at 60 °C, and a melting curve analysis step was added following the manufacturer’s protocol. The cycle threshold (Ct), standard deviation (SD), and coefficient of variation (CV) were obtained for each sample. Ct means were used to assess the gene stability performed with the automated pipeline endoGenes (https://hanielcedraz.shinyapps.io/endoGenes/, accessed on 6 April 2024). This pipeline uses the three most known programs to evaluate gene stability: geNorm [36], NormFinder [37], and BestKeeper [38], generating a final ranking in the RankAgreeg package [39] with the most stable genes. The *RPL5* and *RPL30* genes were selected as the most stable genes found for kidney tissues (Supplementary Table S2).

### 2.6. Primer Design of Target Genes and Gene Expression Analysis

Primers for the eight candidate genes (*CYP24A1*, *FGF3R*, *PTH*, *TRPV6*, *CALB1*, *CALM2*, *FGF1*, and *TRPC3*) were designed using the PRIMER BLAST program [45] with sequences downloaded from the NCBI (https://www.ncbi.nlm.nih.gov/gene, accessed on 6 April 2022) and Ensembl (ensembl.org, accessed on 10 May 2022) databases (Table 2). For the qPCR reactions, 1X of Master MIX SYBR Green 2X, 0.133 uM of forward primer and 0.133 uM reverse primer, cDNA in 1:10 dilution, and ultrapure water (Nuclease Free Water, Qiagen) were used. qPCR reactions were performed in duplicate in 15 µL of the final volume. Subsequently, qPCR reactions were submitted to the QuantStudio 6 Real-Time PCR equipment (Applied Biosystems, Foster City, CA, USA). The reaction conditions to assess the specificities of the amplification were 95 °C for 2 min and 40 cycles for 15 s at 95 °C and 30 s at 60 °C. The expression level of target genes was determined using Ct values, and changes in gene expression were calculated with the 2^−ΔCt^ method [46], using the geometric mean of the endogenous genes *RPL5* and *RPL30*.

### 2.7. Statistical Analysis

To verify differences among groups for performance, egg quality, and bone-related traits, an ANOVA was carried out with the GLM procedure of SAS [48], including the fixed effects of diet and block. The Tukey test was used for mean group comparison, and differences were considered significant if *p* ≤ 0.05. Statistical analyses of qPCR gene expression profiles were performed using the Kruskal–Wallis test, followed by the post hoc Dunn’s test in the R environment [49]. Genes were considered differentially expressed (DE) among the studied groups if the adjusted *p*-value (*p.adj*) was ≤0.05.

## 3. Results

### 3.1. Phenotypic Traits

Of the 22 performance and egg quality traits evaluated in our study, 11 had significant differences among the groups (Table 3). The laying hens receiving the high Ca/P ratio diet had better performance than those receiving the MR diet for egg mass (per hen housed), egg number (per hen housed), and intact eggs. When the comparison was performed with hens receiving the LR diet, the HR group had higher egg yolk color, egg density, eggshell thickness, eggshell (% and g), eggshell strength, and yolk (g) values than the LR group.

### 3.2. Kidney Histopathologic Analysis

**Figure 1:**
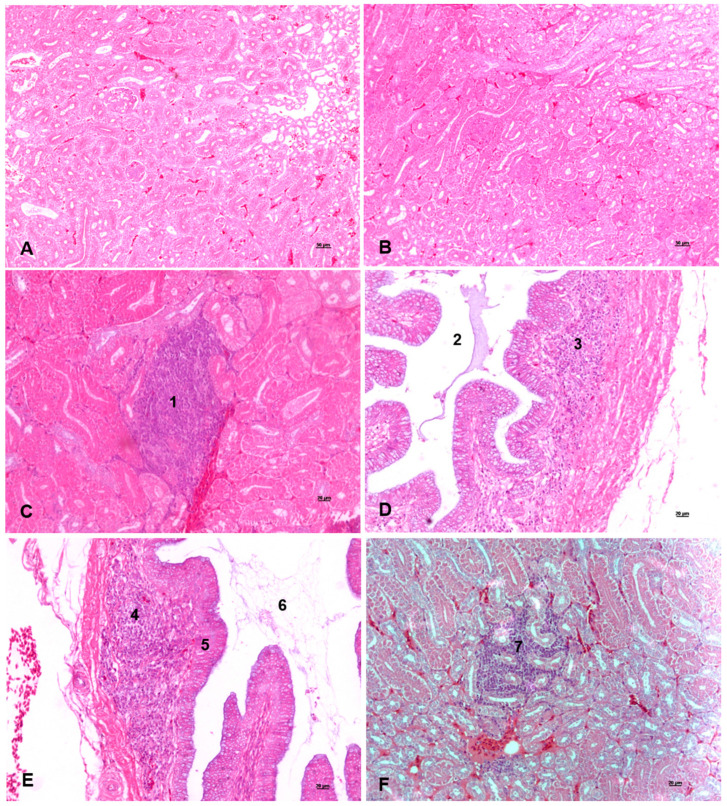
Kidney histopathology stained with hematoxylin and eosin (HE). (**A**,**B**) Normal histology, with no histopathological changes in samples from the high Ca/P ratio diet group. (**C**,**D**) Samples from the medium Ca/P ratio group with mild lymphocytic interstitial infiltration (1), collecting tubule lumen (2), and lymphocytic infiltrate in the collecting tubule mucosa (3). (**E**,**F**) Samples from the low Ca/P ratio group with lymphocytic infiltration in the collecting tubule mucosa (4), collecting tubule lumen (5), collecting tubule epithelial cells (6), and mild interstitial lymphocytic infiltration in the renal cortex (7).

No alterations in the kidney microscopic evaluation were observed in the HR group (Figure 1A,B). In the other two groups, the presence of lymphocytic inflammatory infiltration was observed in 20% of the samples (2 out of 10 animals) from the MR group (Figure 1C,D) and 40% of the samples (4 out of 10 animals) from the LR group (Figure 1E,F).

### 3.3. Tibia and Humerus Morphological and Physical Analysis

There was no difference in the hens’ body weight at 73 weeks of age among the three groups evaluated (*p* > 0.05, Table 4). The only significant difference among the groups for the morphological and physical traits was in the humerus length, which was longer when the hens received an MR diet compared with those fed with an HR diet *(p* < 0.05, Table 4).

### 3.4. Gene Expression Analysis

**Figure 2:**
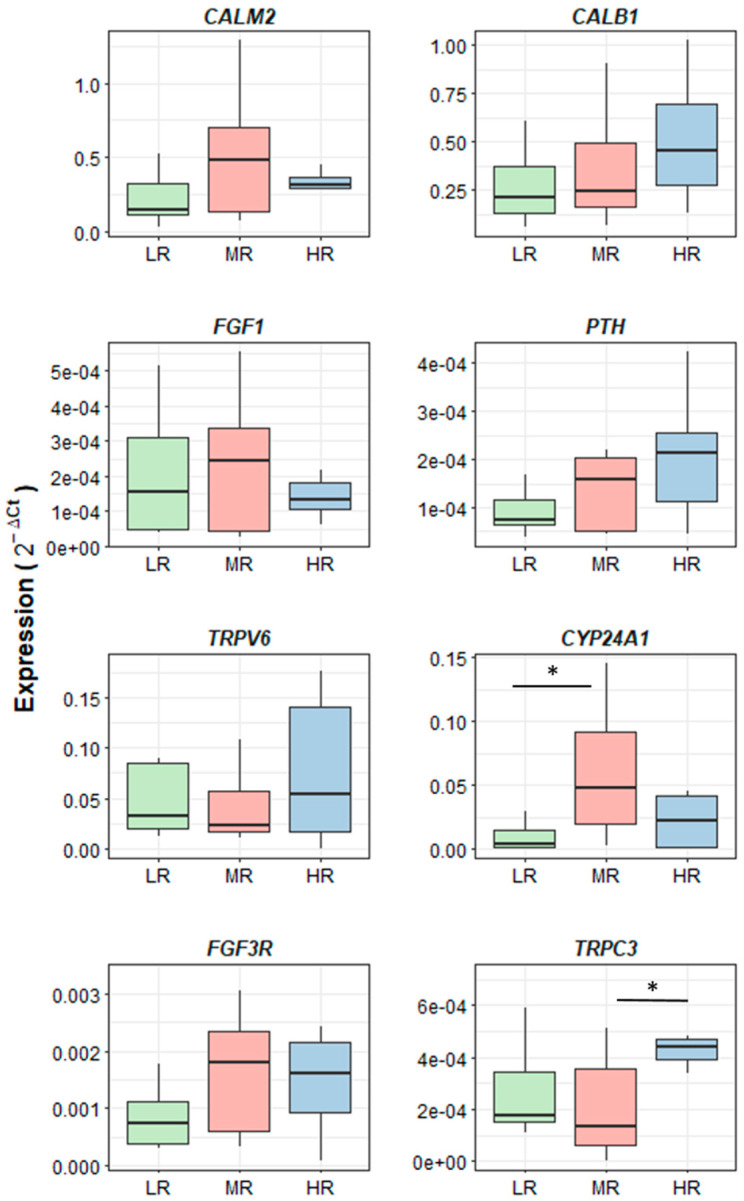
Relative expression of candidate genes performed in kidney of 73-week-old laying hens fed diets with different Ca/P ratios. HR: high Ca/P ratio (22.43 Ca/P), LR: low Ca/P ratio (6.71 Ca/P ratio), MR: medium Ca/P ratio (11.43 Ca/P ratio). * *p.adj* ≤ 0.05. The Figure was prepared using the ggplot2 package from R.

All the evaluated genes were expressed in the kidney tissue, and the Cts varied from 15.65 to 37.39. Of the eight target genes analyzed, two were DE between groups. The *CYP24A1* gene was upregulated by about 2.5-fold (*p.adj* < 0.05, Supplementary Table S3) in the MR compared to the LR group (Figure 2), and the *TRPC3* gene was about four times more expressed in the HR than in the MR group (*p.adj* ≤ 0.05).

## 4. Discussion

The levels of Ca and P added to diets are critical factors for the absorption and metabolism of both minerals [50]. An inadequate Ca:P ratio in the diet can interfere with the availability of these minerals and lead to an imbalance in homeostasis and, consequently, to inadequate bone development and poor eggshell formation, reducing the productive life of laying hens [51].

In general, to maintain daily energy requirements, laying hens have the ability to adjust feed consumption according to the energy level of the diet [52]. In our trial, the hens received isoenergetic feed, resulting in similar feed intake between the evaluated groups (Table 3). However, due to its essential role in egg formation, a different Ca/P ratio significantly affects egg production and egg quality-related traits [53]. Thus, as expected, we observed significant effects on intact eggs (per hen housed and per hen/day), egg density, and eggshell % and g, thickness, and strength, confirming better results for the group that received the diet with the highest Ca/P ratio, since the hens produced eggs with more resistant shells.

Regarding the bone traits, the humerus length was slightly shorter in the HR group than in the MR group (Table 4). Ca and P levels in the diet are essential to bone mineralization [54,55,56] and health [21], and although there was a variation in the humerus length, there was no difference in the other bone traits evaluated. These results indicate that the bone quality traits evaluated in our study were not affected by the different Ca/P ratios provided in the diets among the groups. Probably, the Ca and P levels used in the diets were enough for the laying hens to maintain the minimal requirements for bone quality until older ages. Similar results evaluating bone traits were found when diets with different levels of Ca and P and vitamin D supplementation were evaluated in 57-week-old laying hens [21] and when 42-day-old broilers received diets with different formulations of Ca and non-phytate P [57]. Furthermore, when histological analyses were performed in kidneys, a key organ in mineral regulation, no lesions were found in the HR samples (Figure 1A,B). Therefore, a high Ca/P ratio could be used in the diet of laying hens to reduce costs to poultry producers due to the elevated prices and limited availability of P, without compromising performance, egg quality, and bone integrity traits.

With advancing age, there is a reduction in the digestibility of calcium in the gut [58], and the role of the kidneys in the reabsorption and excretion of this mineral is essential. Since the kidneys are key organs responsible for the correct metabolism of Ca and P [59], we evaluated a set of genes involved in Ca and P regulation to understand and explore their effects in laying hens at their late phase of production. Of the eight candidate genes evaluated in our study, two (*TRPC3* and *CYP24A1*) were DE in the kidneys of laying hens fed diets with different Ca/P ratios.

The *TRPC3* gene encodes a protein that acts mainly in the formation of the non-selective calcium channel and other ions and is activated in response to calcium and influx signaling [60]. The *TRPC3* gene can be activated by the renin–angiotensin system and the amount of fluid within blood vessels to control blood pressure [61]. In humans, *TRPC3* was found to be upregulated in the kidneys of patients with hypertension [62]. Studies carried out in rat fibroblasts found that changes in calcium dynamics coincide with the *TRPC3* expression of calcium channel activity, which is essential to calcium homeostasis [63,64]. Moreover, some members of the TRPC family in calcium channels of the cell membrane are activated by the calcium store depletion mechanism [64,65]. The *TRPC3* gene was upregulated in the HR group when compared to the MR group. Studies have found that the existence of a coupled luminal membrane in the kidney proximal tubules (Tbs) is dependent on *TRPC3* expression. These tubules are located in the Henle loop, which are considered sites of high calcium absorption [66]. Furthermore, in a comparative study, TRPC3 knockout in mice showed deficiency in Tb calcium entry and transport [67]. The signaling in the Tb region indicates that TRPC3 protein is involved in transcellular transport of calcium through Tbs [68]. In this way, the upregulation of *TRPC3* in the HR group may indicate the existing transcellular activity in the epithelial calcium transport in laying hens at their late phase of production. This could be occurring because the HR group received the highest level of Ca in its diet and, consequently, had higher Ca reabsorption [69] rates, which could be beneficial to improve egg quality traits, such as those related to eggshell resistance and intact eggs (Table 3).

The *CYP24A1* gene encodes a member of the cytochrome family that has a role in Ca and vitamin D homeostasis by promoting vitamin D catabolism and reducing the circulating 25-hydroxyvitamin D3 and 1α,25-(OH)2 D3 [70]. In our study, *CYP24A1* was upregulated in the MR group when compared with the LR group. In humans, mutations in this gene have been associated with hypercalcemia, hypercalciuria, hypophosphatemia [71], and idiopathic infantile hypercalcemia [72]. In laying hens, some studies evaluated the *CYP24A1* gene to verify its role in osteogenesis [73] and in the regulation of serum vitamin D [31,74]. Gloux et al. [31] observed that kidney *CYP24A1* upregulation occurred earlier in old than in young hens at 18–19 h post ovulation, attributing this result to the need to stimulate Ca retention and bone resorption. In our study, the hens receiving high Ca levels (HR and MR) did not differ between them in *CYP24A1* expression, while this gene was 2.5 times more expressed in the hens fed with the MR diet than those fed the LR diet. Thus, high levels of Ca in the diet could help to control Ca in the cells through compensatory mechanisms and active transport and, consequently, improve egg density and eggshell thickness, percentage, and weight (Table 3), enlarging the laying hens’ productive life with good-quality eggs.

For the other genes evaluated in the current study (*PTH, CALB1, FGF3R, FGF1*, and *CALM2*), no differential expression was observed among the groups. Although these genes are important for Ca and P regulation in the cell, the different levels of Ca/P provided in the diet did not influence their expression profile at 73 weeks of age, possibly because none of the levels used were considered deficient in the hens. Furthermore, these results could also be explained by diet adaptation since the laying hens received the same feed for a long time, indicating a compensation mechanism to maintain calcium absorption [75]. Probably, most of these genes would be differentially regulated in early ages when mineral renal physiology is more active [16]. Finally, when analyzing the gene expression profile, it was possible to observe that, for most of the genes, the MR group had a higher variance compared to the other two groups, due to the high individual variation in this group, which could affect the detection of differentially expressed genes.

In the last decade, studies using molecular biology have been conducted to help understand mineral metabolism in laying hens, especially comparing the levels of gene expression among different ages [6,7,23,76]. However, there is a lack of information on how hens in the late laying period respond to different Ca and P ratios in their diet, as well as the role of the analyzed genes and their expression profile in the kidneys. In our study, we found the *CYP24A1* and *TRPC3* genes differentially expressed in the kidneys of 73-week-old laying hens. The expression profile found could be involved in calcium and phosphate transcellular transport in laying hens at their late phase of production and as a way to keep mineral absorption at adequate levels to meet metabolic requirements, as suggested by Vargas et al. [77]. In this way, it is interesting to note that both differentially expressed genes differed between the MR and the LR or HR groups, which shows that while *TRPC3* expression increased with high levels of Ca/P, a different pattern was observed for *CYP24A1*, highlighting the importance of the Ca/P ratio in the regulation of gene expression. These expression profiles could favor mineral reabsorption, helping to maintain the high egg production and egg quality traits. Furthermore, despite the differences in mineral levels, the expression of important functional genes, such as *CALB1* and *CALM2*, remained unchanged, indicating the existence of mechanisms of compensation and adaptation to mineral utilization in chickens. This could help to mediate poultry health, mainly through mechanisms of long-term adaptive capacity, as has been suggested in broilers [75]. Finally, since mineral regulation mechanisms are not well described in laying hens’ kidneys at molecular levels, our findings highlight the expression profiles of candidate genes in hens with advanced age, as well as their possible influence on production traits.

## 5. Conclusions

A high ratio of Ca:P provided in the diet of laying hens improved egg production and egg quality traits, especially those related to eggshell resistance. The *CYP24A1* and *TRPC3* genes were differentially expressed among 73-week-old laying hens receiving diets with different Ca:P ratios, which could facilitate the calcium and phosphate absorption, helping to maintain the bone quality and performance traits of the laying hens in this late phase of production.

## Figures and Tables

**Table 1:** Primers for the reference candidate genes evaluated in kidneys of laying hens.

Gene	Gene ID	Primers (5′-3′)
*RPL30* ^1^	ENSGALG00000029897	F: 5′-ATGATTCGGCAAGGCAAAGC-3′
(Ribosomal Protein L30)		R: 5′- GTCAGAGTCACCTGGGTCAA-3′
*RPL4* ^1^	ENSGALG00000174444	F: 5′-TGTTTGCCCCAACCAAGACT-3′
(Ribosomal Protein L4)		R: 5′-CTCCTCAATGCGGTGACCTT-3
*HBMS* ^2^	ENSGALG00000042939	F: 5′-ACTAGTTCACTTCGGCGAGC-3′
(Hydroxymethylbilane Synthase)		R: 5′-CTCAGGAGCTGACCTATGCG-3′
*GAPDH* ^3^	ENSGALG00000014442	F: 5′-TGGGAAGCTTACTGGAATGG-3′
(Glyceraldehyde 3 Phosphate Dehydrogenase)		R: 5′-ATCAGCAGCAGCCTTCACTAC-3′
*MRPS30* ^4^	ENSGALG00000014874	F: 5′-CCTGAATCCCGAGGTTAACTATT-3′
(Mitochondrial Ribosomal Protein S30)		R: GAGGTGCGGCTTATCATCTATC-3′
*MRPS27* ^4^	ENSGALG00000015002	F: 5′-GCTCCCAGCTCTATGGTTATG-3′
(Mitochondrial Ribosomal Protein S27)		R: 5′-ATCACCTGCAAGGCTCTATTT-3′
*HPRT1* ^3^	ENSGALG000000006098	F: 5′-TGGGGATGACCTCTCAACCT-3′
(Hypoxanthine Phosphoribosyltransferase 1)		R: 5′-TCCAACAAAGTCTGGCCGAT-3′
*RPLP1* ^3^	ENSGALG00000030878	F: 5′-CCCTCATTCTCCACGACGAC-3′
(Ribosomal Protein Lateral Stalk Subunit P1)		R: 5′-CCAGAGCCTTAGCAAAGAGAC-3′
*RPL5* ^4^	ENSGALG00000005922	F: 5′-AATATAACGCCTGATGGGATGG-3′
(Ribosomal Protein L5)		R: 5′-CTTGACTTCTCTCTTGGGTTTCT-3′
*TOP2B* ^5^	NM_205082.1	F: 5′AAGGCCAAGAAGATGGAAACTG3′
(DNA Topoisomerase II Beta)		R:5′TCTTGGATTTCTTGCATGGTGT3′

^1^ Petry et al. (2018) [40], ^2^ Paludo et al. (2016) [41], ^3^ Marciano et al. (2020) [42], ^4^ Nascimento et al. (2015) [43], ^5^ Okino et al. (2015) [44].

**Table 2:** Primers for the target genes evaluated in kidneys of laying hens.

Gene	Ensembl ID	Primer Sequence (5′-3′)	Amplicon Size (bp)
*CYP24A1*	ENSGAL00000042279	F: 5′-GCCCTGTGCTGGATCATTCG-3′	142
(Cytochrome P450 family 24 subfamily A member 1)		R: 5′-GCCGTCATTAGTCAAGCTGC-3′	
*FGFR3*	ENSGAL00000015708	F: 5′-CCACTCAAGAGACAGGTAACAGTG-3′	179
(Member of the basic fibroblast growth factor)		R: 5′-CCCAGGGTCAGGCGAGAA-3′	
*PTH*	ENSGAL00000061928	F: 5′-CCATCTGCTGACATACCCCAA-3′	158
(Parathyroid hormone)		R: 5′-TCACTCACCGATCTCTTCATCATTG-3′	
*TRPV6*	ENSGAL00000014746	F: 5′-TGGAGAGCCCAGATTGTTGC-3′	114
(Transient receptor potential cation channel subfamily V member 6)		R: 5′-ATACCATCGGTCCCCTAGCC-3′	
*FGF1* ^1^	ENSGALG00000007343	F: 5′-CTGTATGGCTCGCAGCTACC-3′ R: 5′-CTGTTCCCGTTTTTCTTCAGCC-3′	134
(Fibroblast growth factor 1)			
*TRPC3*	ENSGALG00000011875	F: 5′- CGTGTAGCAGGCTTGGAAGA-3′	92
(Transient receptor potential cation channel subfamily C member 3)		R: 5′-CACCAGCAGGCCTAGGAAAA-3′	
*CALB1* ^2^ (Calbindin 1)	ENSGALG00000015914	F: 5′-GGCAGGCTTGGACTTAACAC-3′ R: 5′-GCTGCTGGCACCTAAAGAAC-3′	143
*CALM2* ^2^ (Calmodumin 2)	ENSGALG00000010023	F: 5′-CCACCATGGCTGATCAACTG-3′ R: 5′-GCCATTGCCATCAGCGTCTA-3′	192

^1^ Marchesi et al. (2021) [47], ^2^ Paludo et al. (2017) [41].

**Table 3:** Means and standard errors for performance and egg quality traits evaluated in laying hens fed diets with different Ca/P ratios.

Traits	LR	MR	HR	ProbF
Albumen height, mm	7.739 ± 0.092	7.855 ± 0.085	7.630 ± 0.102	0.2075
Feed intake, g	107.0 ± 1.79	106.8 ± 2.07	106.9 ± 1.59	0.9979
Feed conversion rate (per dz of eggs)	1.335 ± 0.021	1.371 ± 0.019	1.338 ± 0.013	0.0945
Feed conversion rate (per egg mass)	1.767 ± 0.019	1.805 ± 0.021	1.754 ± 0.017	0.1027
Color	4.223 ± 0.060 b	4.507 ± 0.052 a	4.626 ± 0.058 a	<0.0001
Density, g/ml	1083.1 ± 0.44 b	1086.4 ± 0.51 a	1087.7 ± 0.40 a	<0.0001
Shell thickness, mm	0.364 ± 0.003 b	0.378 ± 0.003 a	0.380 ± 0.002 a	0.0008
Egg mass (per hen housed)	57.05 ± 2.13 ab	55.33 ± 1.60 b	60.64 ± 0.84 a	0.0284
Egg mass (per hen/day)	60.54 ± 0.69	59.34 ± 1.08	61.65 ± 0.52	0.1576
Egg number (per hen housed)	90.73 ± 3.17 ab	87.69 ± 2.33 b	95.45 ± 1.24 a	0.0140
Egg number (per hen/day)	96.21 ± 0.22	93.93 ± 1.53	97.01 ± 0.36	0.0535
Egg weight, g	62.87 ± 0.61	63.24 ± 0.36	63.81 ± 0.45	0.3896
Intact eggs (per hen housed)	89.83 ± 3.10 ab	86.82 ± 2.31 b	94.63 ± 1.29 a	0.0113
Intact eggs (per hen/day)	95.28 ± 0.24 ab	93.01 ± 1.55 b	96.18 ± 0.42 a	0.0521
Eggshell, %	9.162 ± 0.069 b	9.604 ± 0.027 a	9.513 ± 0.074 a	0.0002
Eggshell, g	5.714 ± 0.057 b	6.014 ± 0.021 a	6.007 ± 0.053 a	0.0007
Eggshell strength, Kgf	4.030 ± 0.081 b	4.243 ± 0.080 ab	4.458 ± 0.069 a	0.0134
Albumen weight, g	39.80 ± 0.35	39.74 ± 0.14	40.01 ± 0.47	0.8505
Albumen, %	63.82 ± 0.20	63.39 ± 0.16	63.25 ± 0.32	0.0958
Yolk, %	27.02 ± 0.17	27.00 ± 0.16	27.24 ± 0.31	0.6120
Yolk, g	16.85 ± 0.13 b	16.93 ± 0.15 ab	17.23 ± 0.15 a	0.0464
Haugh unit	87.03 ± 0.54	87.65 ± 0.58	86.32 ± 0.59	0.1630

HR: high Ca/P ratio (22.43 Ca/P), LR: low Ca/P ratio (6.71 Ca/P ratio), MR: medium Ca/P ratio (11.43 Ca/P ratio). Different letters in the same line indicate statistical differences among the evaluated groups (*p* < 0.05).

**Table 4:** Means and standard errors of phenotypic traits evaluated in tibia and humerus bones from laying hens fed diets with different Ca/P ratios.

Bone Traits	Groups			ProbF
	LR	MR	HR	
Body weight (g)	1728.9 ± 64.4	1836.5 ± 65.7	1836.3 ± 77.6	0.3047
Tibia weight (g)	8.261 ± 0.144	8.550 ± 0.179	8.165 ± 0.192	0.1161
Tibia length (mm)	116.1 ± 0.91	117.6 ± 0.76	116.4 ± 1.22	0.2914
Tibia BS (kgf)	15.13 ± 1.33	17.67 ± 1.15	14.58 ± 0.91	0.2462
Tibia FLEX (kgf/mm)	11.80 ± 0.99	12.53 ± 0.75	12.60 ± 1.09	0.7267
Seedor Index (mg/mm)	0.071 ± 0.001	0.075 ± 0.002	0.073 ± 0.003	0.3890
Humerus weight (g)	3.572 ± 0.218	3.485 ± 0.186	3.235 ± 0.363	0.8351
Humerus length (mm)	74.06 ± 0.59 ^ab^	75.69 ± 0.56 ^b^	73.23 ± 0.81 ^a^	0.0193

HR: high Ca/P ratio (22.43 Ca/P), LR: low Ca/P ratio (6.71 Ca/P ratio), MR: medium Ca/P ratio (11.43 Ca/P ratio). Different letters in the same line indicate statistical differences among the evaluated groups (*p* < 0.05).

## Data Availability

All data are available in the article.

## Supplementary Materials

The following supporting information can be downloaded at: https://www.mdpi.com/article/10.3390/ani14101407/s1, Table S1: Formulated diet for the three studied groups (HR, LR, MR), Table S2: Gene ranking showing the most stable genes among different tools evaluated, Table S3: Results of qPCR statistical analysis of gene expression using Kruskal–Wallis test considering each comparison of the studied groups.
